# Blink-To-Live eye-based communication system for users with speech impairments

**DOI:** 10.1038/s41598-023-34310-9

**Published:** 2023-05-17

**Authors:** Mohamed Ezzat, Mohamed Maged, Youssef Gamal, Mustafa Adel, Mohammed Alrahmawy, Sara El-Metwally

**Affiliations:** Department of Computer Science, Faculty of Computers and Information, Mansoura University, P.O. Box: 35516, Mansoura, Egypt

**Keywords:** Computer science, Information technology, Software

## Abstract

Eye-based communication languages such as Blink-To-Speak play a key role in expressing the needs and emotions of patients with motor neuron disorders. Most invented eye-based tracking systems are complex and not affordable in low-income countries. Blink-To-Live is an eye-tracking system based on a modified Blink-To-Speak language and computer vision for patients with speech impairments. A mobile phone camera tracks the patient’s eyes by sending real-time video frames to computer vision modules for facial landmarks detection, eye identification and tracking. There are four defined key alphabets in the Blink-To-Live eye-based communication language: Left, Right, Up, and Blink. These eye gestures encode more than 60 daily life commands expressed by a sequence of three eye movement states. Once the eye gestures encoded sentences are generated, the translation module will display the phrases in the patient’s native speech on the phone screen, and the synthesized voice can be heard. A prototype of the Blink-To-Live system is evaluated using normal cases with different demographic characteristics. Unlike the other sensor-based eye-tracking systems, Blink-To-Live is simple, flexible, and cost-efficient, with no dependency on specific software or hardware requirements. The software and its source are available from the GitHub repository (https://github.com/ZW01f/Blink-To-Live).

## Introduction

Amyotrophic Lateral Sclerosis, ALS, and Primary Lateral Sclerosis, PLS are progressive neuron diseases that affect the brain and spinal cord cells and gradually cause the loss of muscle control and develop speech impairment symptoms. Patients can communicate with their caregivers in the later disease stages through eye gestures^1,2^. Translating eye gestures into a communicated speech invented a plethora of Augmentative/Alternative Communication (AAC) devices that have different designs and usability concepts ranging from control panels with letters and numbers, touch and gaze-sensing screens, eye tracking systems and consequently modified mouse cursor techniques are introduced to control different computer applications. Commercial gaze-sensing keyboards are very expensive; for example, Tobii Dyanvox^3^ has a cost ranging from 5K$ to 10K$ according to different configuration models. Eye Transfer^4^ (E-tran) board is an alternative low-cost solution ($260) where a caregiver holds a transparent plastic board of printed letters and observes the patient’s eye gestures on the board. The head-mounted eye gaze trackers^5^ required some static, adjusted settings according to the camera and patient’s eye during the head movement.

Thoughts and intentions are another communication approach for patients with speech impairments. Brain-Computer Interface (BCI) is utilized brain activity (i.e., EEG signals) to control external devices, such as typing words by selecting letters on a digital keyboard^6^ or performing complex tasks such as browsing a web page^7^ or painting an image^8^. Some brain spellers have different communication rates^9,10^, which increased recently by combining the language model and deep learning^11,12^. Research studies stated that most ALS/PLS patients have a good acceptance rate of using technologies based on eye tracking to initiate communications with their surrounding world^13–15^, and tracking the patient’s eyes is much simpler than tracking or detecting other signals, such as EEG/ECG^16,17^.

Patients with speech impairments lose their natural speaking abilities^18^. Accordingly, many modified speaking languages that utilize the available moving organs such as the head, facial gestures, eyes, or brain signals are proposed^19,20^. Eye-based communication languages are introduced in different forms encoding different eye gestures to easily and efficiently synthesize a communicated speech^21–23^. Morse code is one of the proposed approaches for encoding the short and long eye blinks as a sequence of dots and dashes, and the alphabets/sentences are constructed accordingly^22,23^. Blink-To-Speak is another proposed eye language with eight alphabets according to eight eye gestures (Shut, Blink, Left, Right, Up, Down, Wink, and Roll). The most daily life phrases (i.e., 50 commands) are encoded using these defined eye alphabets in an online book in different native languages to train and teach the patients/caregivers how to use the language easily and efficiently^24^. Most of the proposed eye languages for speech impairments are implemented in specialized hardware devices with specific sensors (eyeglasses with infrared, eye gaze keyboards, head-mounted eye trackers, etc.) that complicate the communication process with less usability and accessibility for the patients and caregivers^14,25,26^. Also, some of these devices are expensive and not affordable in low-income counties such as Egypt.

Further, the eye blinking is the only considered state in the previously proposed eye language, which can limit the application’s usability by discarding other eye gestures (i.e., left, up, right, etc.) that can encode more different phrases and daily life commands^14,22,23^. While Blink-To-Speak eye language has more alphabets than other eye-based communication languages, which can encode more phrases, the caregivers can only understand the patient’s intended speech by observing their eyes, and the patients/caregivers are trained through a manual book to speak/understand the language. Also, the defined communicated sentences in the book may have a long sequence of different eye gestures that need more training time and can exhaust the patient’s eyes.

In this paper, we proposed Blink-To-Live, an eye-tracking system based on a modified Blink-To-Speak language for patients with speech impairments. A handheld mobile device with supported cameras captures real-time video frames and sends them to computer vision modules for facial landmarks detection, eye identification and tracking. The Blink-To-Live communication system has four eye movements: left, right, up, and blink. These eye gestures encode more than 60 daily life commands expressed by a sequence of three eye movements. The speech generation and translation modules decode the recognized eye movements into corresponding phrases, display them on the mobile screen in a patient’s native speech, and their synthesized voice can be heard accordingly. Unlike the other sensor-based eye-tracking systems, Blink-To-Live is simple, flexible, and cost-efficient, with no dependency on specific software or hardware requirements. Also, compared to the original Blink-To-Speak, the proposed eye-based communication language has more commands with a short sequence of eye movements to increase its usability for the patient to speak faster in less training time.

Our paper is organized as follows: Section “Related work” provides a summary of the previously related work, Section “Materials and methods” presents the big picture of our proposed system architecture with its internal modules and sub-modules that are discussed in detail in the following subsections, Section “Experimental results” demonstrates the main experimental results of our proposed communication system including different user interfaces, basic services provided to patients/caregivers, and different usability and accessibility issues, Section “Conclusion” concludes the paper and highlights some future insights for improving the Blink-To-Live system.

## Related work

**Figure 1:**
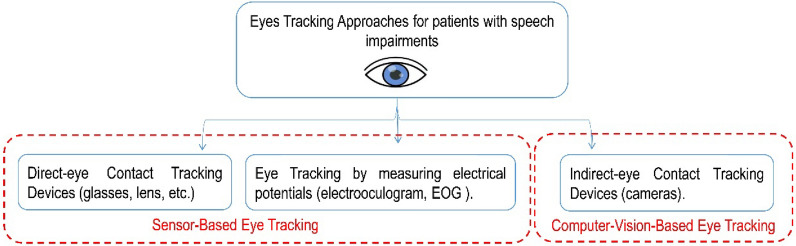
Eye-tracking systems for patients with speech impairments.

Eye tracking technologies are utilized in many sciences such as cognition, psychology, computer, and medicine to digitize how people interact with their living environment^27,28^. Tracking human eyes, which implies recording their different movements, is essential for many pervasive applications such as eye-based communications and computer environment interactions^29,30^. Three types of eye-tracking approaches were introduced previously (see Fig. 1): one relies on devices attached to the eyes directly, such as special lenses or glasses with infrared sensors that track and record eye positions. The second approach targets the eyes as a source of electrical field that can be detected in the darkness even when the eye is closed and measures the electrical potentials by positioning some electrodes around human eyes, the example of this approach is the electrooculogram (EOG) technique^22,27^. Since the previous two mentioned approaches rely on some sensors such as infrared or electrodes, they can be grouped under the term sensor-based eye tracking technologies. The other eye-tracking methods rely on computer-vision techniques for detecting and tracking the human eyes in the captured video frames by a camera in real-time without direct contact with human eyes or using extra hardware sensors^31–33^.

One proposed human–computer interaction method is positioning nearly five electrodes around the patients’ eyes to detect their blinks and movements to control the cursor and different desktop applications^34,35^.‏ ‏Some applications have a user interface with a keyboard to enable the patients to use their eyes to look at the intended letters and select based on eye blinks and movements for a predefined time slot (i.e., one second). Also, electromyography systems can detect facial muscle signals and control the mouse cursor and computer applications accordingly^36^. The eye gestures and signals from facial muscles are incorporated to increase the reliability of applications that support people with speech impairments in communication and cursor control. While combining these two techniques has high accuracy, it still suffers from a slow communication speed and high cost^22^. Direct-eye contact approach targets designing hardware devices such as eyeglasses with an infrared sensor that translates eye blinks into a sequence of alphabets using a Morse code with variations that the alphabets are displayed on the LCD screen^23^, or the synthesized phrases are displayed on a mobile phone screen^22^. EyeLive^37^ is another eye-tracking system based on measuring eye reflections by the infrared sensor. The system has a user interface with a keyboard to let the patient selects the intended letters using their eye gestures. While the infrared sensor facilitates eye gesture detection by increasing the reflection light from the eyes, it has some limitations, such as interference with sunlight in outdoor usage, and the relative eye positioning for the infrared sensor will affect the tracking performance. The direct-eye contact approaches have an extra cost of using external hardware devices for eye tracking with less reliability and robustness according to inaccurate sensor measurements.

Some machine learning techniques are introduced to detect and track the human eyes, such as principal component analysis, which detects the first eye’s six principal components in the captured images. The eye’s pupil position is classified using the artificial neural network model^38^. The system required a special hardware configuration such as a head-mounted camera and wearing glasses that complicate the communication process. Some machine learning models are used to predict the eye’s directions, gaze angles, and movements, such as Adaptive Linear Regression, ALR^39^, K Nearest Neighbors, KNN^40^, and Random Forest, RF^41^, which suffer from inaccurate prediction accuracy due to insufficient training samples, parameters setting, noisy images, or complex feature extraction process^31^. Also, Support Vector Machine, SVM, is used to classify the five eye directions after the eye region is detected by modifying the characteristics of the active appearance model^42^. A deep learning Convolution Neural Network, CNN, with multi-layer architecture, is used to predict different eye gestures based on training the CNN with a large number of samples with known eye states as predicted labels, which can achieve good performance but require a large number of training samples, long training time, and tune the model’s parameters accordingly^43^. The eye-tracking technology is applied in non-communication tasks such as understanding the factors that influence comprehension by investigating how developers comprehend query syntax and method syntax in Language-Integrated Query (LINQ), which is a popular technology for querying data in .NET programming languages such as C# and VB.NET^44^. Also, the eye-tracking technology is integrated with Virtual Reality (VR) head-mounted displays for rendering the VR senses, assisting the user interaction with the environment^45^ or supporting the vision screening^46^.

## Materials and methods

Blink-To-Live is a mobile application based on computer vision techniques for patients with motor neuron disorders such as ALS and PLS. These patients gradually develop speech impairment symptoms. In the final paralyzed stage, eye gestures are the only methods to initiate communication. The Blink-To-Live communication system relies on the four eye gestures: Left, Right, Up, and Blink (Table 1), defining the language's key alphabets for encoding more than 60 daily life communicating sentences, such as those presented in Tables 2 and 3.

**Table 1:** Four defined eye alphabets in the Blink-To-Live eye-based communication system.

Eye alphabets	Eye states
Blink	
Left	
Right	
Up

**Table 2:** Encoded sentences by four eye gestures—Part 1 (L: Left, R: Right, U: Up, B: Blink).

Eye states			Eye-movements encoded sentences
L	L	L	Water
L	L	R	Adjust my Specs
L	L	U	Feeling Cold
L	L	B	Feeling Hot
L	R	L	Emergency
L	R	R	Nose Block
L	R	U	Palpitations
L	R	B	I am not Okay
L	U	L	Choking
L	U	R	Heartache
L	U	U	Danger
L	U	B	Dizziness
L	B	L	Headache
L	B	R	Breathless
L	B	U	Cramps
L	B	B	Let’s Talk
R	L	L	I Want to Go Home
R	L	R	Wipe
R	L	U	Wash
R	L	B	Scratch
R	R	L	Change Clothes
R	R	R	Food
R	R	U	Change Position Sitting
R	R	B	Change Position Lay straight
R	U	L	Let’s Go Out in the Open
R	U	R	Open the Door
R	U	U	Open the Window
R	U	B	Television
R	B	L	Music
R	B	R	Light
R	B	U	Air Conditioning
R	B	B	Newspaper

**Table 3:** Encoded Sentences by Four Eye Gestures – Part 2 (L: Left, R: Right, U: Up, B: Blink).

Eye states			Eye-movements encoded sentences
U	L	L	Comb my hair
U	L	R	I Want to Spit
U	L	U	Change Bedsheet or Pillow
U	L	B	Someone is hurting me
U	R	L	Call a Doctor
U	R	R	Take a Shower
U	R	U	Food Stuck in Teeth
U	R	B	I Need a Hug
U	U	L	Call the Police
U	U	R	Call a Relative
U	U	U	Toilet
U	U	B	Change Diaper
U	B	L	My Phone Camera is not Working
U	B	R	My Computer is not Working
U	B	U	Good to See You
U	B	B	My Wheelchair is not Working
B	L	L	No
B	L	R	Hello
B	L	U	Long time no see
B	L	B	I Want to Sleep
B	R	L	I Want to meet my Pet
B	R	R	Yes
B	R	U	I Want to Pray
B	R	B	The Internet is not Working
B	U	L	Congratulation
B	U	R	I am sorry
B	U	U	I love you
B	U	B	How are you
B	B	L	I am in Pain
B	B	R	Medication
B	B	U	Thank You
B	B	B	I am Okay

**Figure 2:**
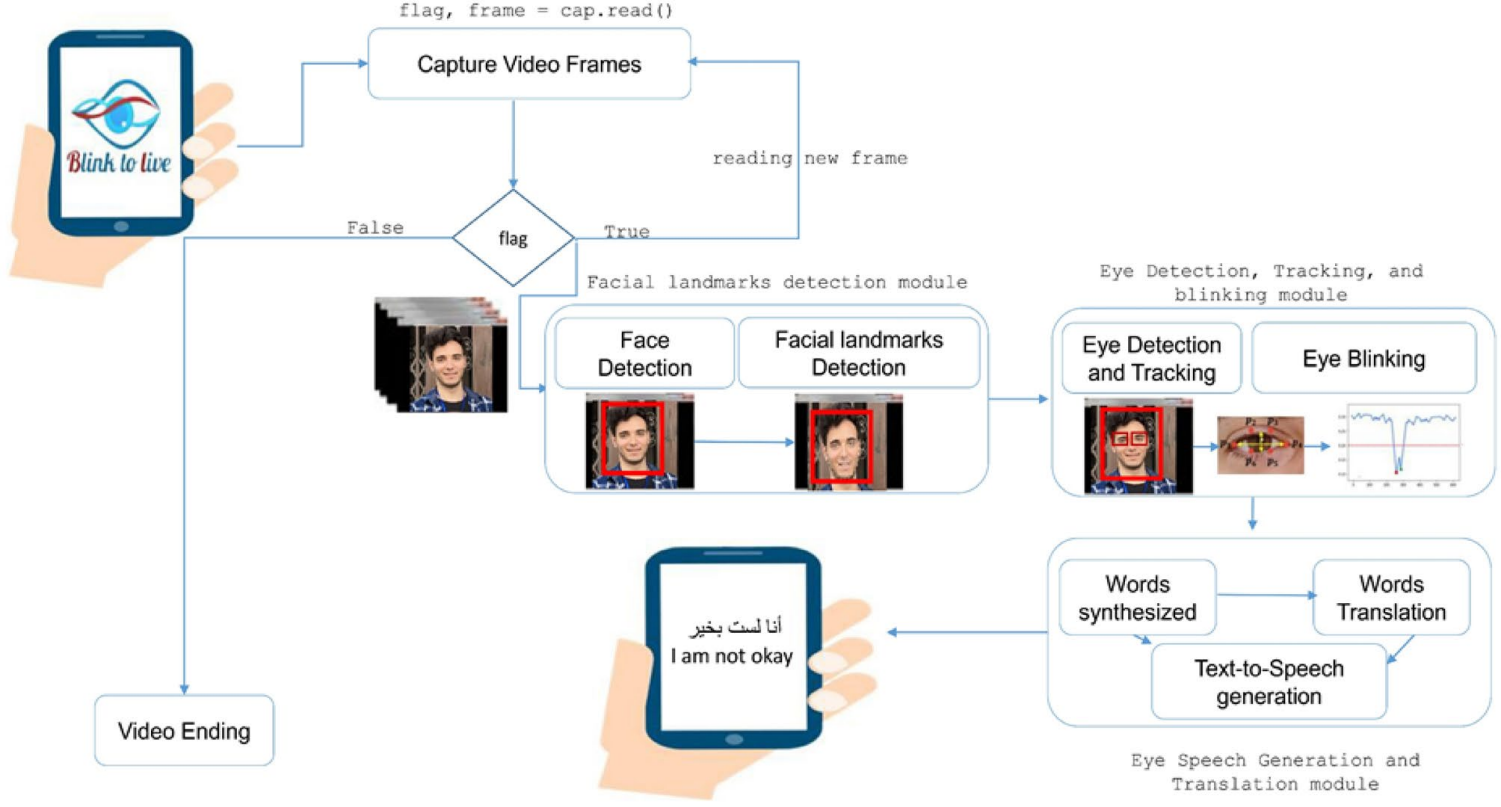
Blink-To-live Communication System Architecture.

As depicted in Fig. 2, Blink-To-Live has two basic system components: a mobile application developed by a Google flutter^47^ framework and the other is a backend python module for video frames image analysis and processing. The patient's interactions with the Blink-To-Live system start with a caregiver's opening the phone camera to track the patient’s eye gestures. Once the video frames are captured correctly in real-time, they are automatically sent to computer vision modules to detect and track the patients’ eye movement states.

The first module is facial landmarks detection, which has two sub-modules: face detection and facial landmarks prediction. This module aims to detect the patient's face and extract the locations of 68 facial coordinates representing different facial structures such as eyes, mouth, nose, etc. The next module detects the patients’ eyes and tracks their movements according to the Blink-To-Live four key alphabets: Left, Right, Up, and Blink. The patient’s communicated speech is generated according to the predefined dictionary of sentences encoded using a combination of three eye movement states. Once the sentences are synthesized, a translation module displays the phrases in the patient’s native language, such as Arabic, German, etc. Also, the text-to-speech module generates a corresponding lifelike speech that helps the patients to communicate easily and efficiently. The detailed implementations of each module will be discussed in the following sections.

### Blink-To-Live eye-based communication language

Blink-To-Live eye-based communication language has four eye alphabets (i.e., Blink (B), Left (L), Right (R), and Up (U)). We modified a previously proposed Blink-To-Speak eye language according to the four eye movements recognized by our system to simplify the patient communication process by expressing more statements with less sequence of eye gestures and transitions. In the Blink-To-Live eye-based communication language, each communicated sentence is expressed as a sequence of three eye states, and each state could be one of the four states (B, L, R, and U). The four defined states will generate exactly 64 daily life-communicated phrases for ALS/PLS patients (Tables 2 and 3). This configuration can be mathematically defined as follows: If you have $n$ recognized eye states and a group of communicated sentences, each sentence will be expressed by $k$ words ($k$ sequence of states), the total number of uniquely communicated sentences will be ${n}^{k}$. By increasing the number of $n$ recognized eye states and the $k$ expressed words, more statements are added to the modified Blink-To-Speak language. We found that when $n=4$, $k=3$, the total number of defined statements equals 64, sufficient to express most of the required daily life communication speech for ALS/PLS patients with less training time. The same eye states (i.e., all three states are left, right, up, or blink) with no transition in between are devoted to the basic patient needs such as food, water, toilet, I am okay, etc. Also, the states with low transitions are dedicated to the most important command, such as changing a diaper, calling a relative, taking medication, etc. Usually, the sequence of the same eye states will be expressed faster by patients and take less training time than the sequence of eye states that transition from one eye state to another. The proposed Blink-To-Live eye-based communication language will be simpler, more flexible, and usable as the patient’s eyes could be exhausted by a long sequence of eye gestures and transitions defined in the original Blink-To-Speak book. Table 4 shows the key differences between Blink-To-Speak^24^ and Blink-To-Live systems.

**Table 4:** The key differences among Blink-To-Speak and Blink-To-Live systems.

Blink-To-Speak	Blink-To-Live
50 commands	64 commands
Manual online book	Desktop and mobile application
Alphabets of eight eye states	Alphabets of four eye states
Communicated sentences have a variable sequence length of words (i.e., eye states) and could be a long sequence of eye states with many transitions	Communicated sentences have a fixed sequence length of words (i.e., three eye states) with fewer transitions
Complex non-automated system	Simple automated system
Long training time	Less training time
Caregiver support is required	Caregiver support is not required after the camera is opened
No priority is given to some sentences compared to others	Sentences expressing the patient’s basic needs are encoded by the same sequence of eye states with fewer transitions to speak quickly
The patient's eyes could be exhausted by expressing long sequence of eye states	The short sequence of eye states might save the patient's eyes from exhaustion

### Facial landmarks detection module

Facial landmarks detect important parts of the face, such as the nose, eyes, eyebrows, mouth, etc. Our system's most important facial structure is the patient’s eyes. The facial landmarks module in our proposed framework has two basic steps: detecting the face from the images collected from video frames and localizing the important facial structures on the face region of interest accordingly.

#### Face detection module

Face detection is accomplished with a pre-trained model called Histogram of Oriented Gradients with Linear SVM (HOG + SVM). HOG descriptor of a human face can be built by dividing the face image into small blocks, and for each block, the gradients (i.e., small changes in the pixel values in terms of x and y directions) are computed. Then, the histogram is generated for each block separately. Finally, the gradient vectors are normalized and combined into a single HOG feature descriptor fed into a linear SVM for face/non-face object classification^48^.

#### Facial landmarks detection module

**Figure 3:**
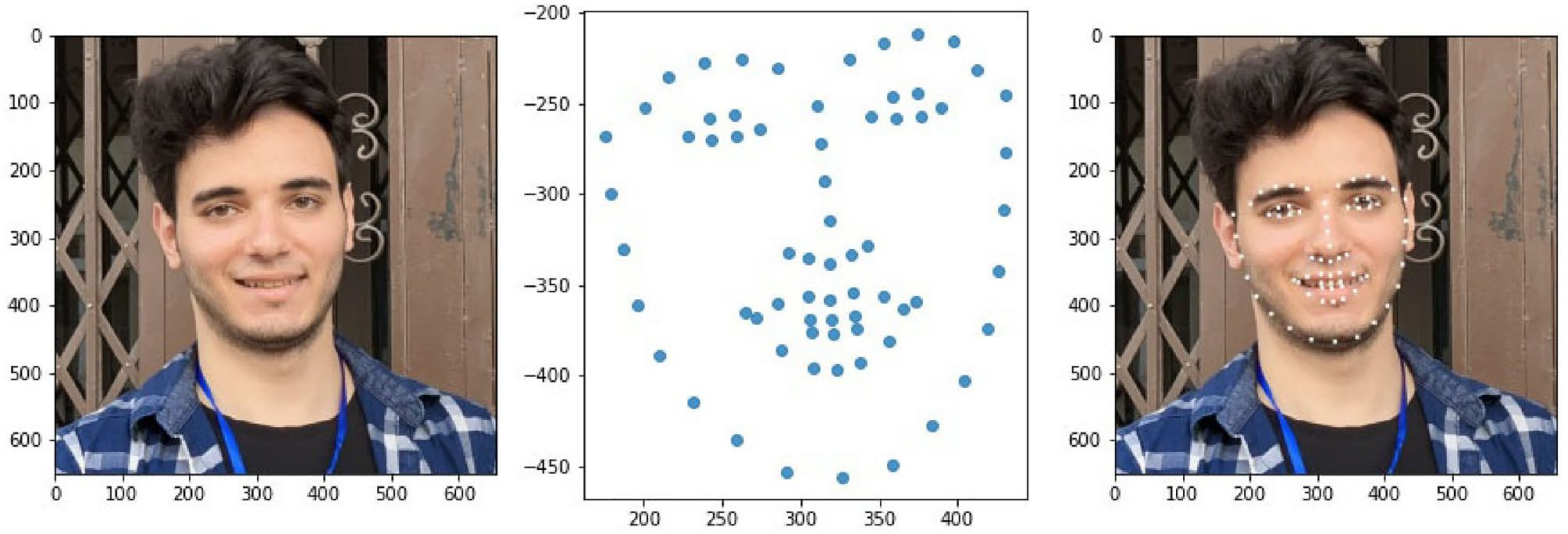
Facial landmarks detection module, where dots represent the 68 coordinates of different facial structures such as mouth, left right eyebrows, nose, etc.

Given that the face region is detected, the next step is detecting the facial landmarks by localizing and labeling the mouth, left and right eyebrows, left and right eyes, nose, and jaw. A set of manually labeled key facial structures in terms of x and y coordinates, along with the pixels’ intensity values and the prior probabilities of the distance of the pixel values corresponding to the facial landmarks pairs, are fed into an ensemble model of regression trees^49^ to train a model for facial landmark detector. The model is implemented in the dlib library^50^ and trained on iBUG 300-W dataset^51^ for estimating the locations of 68 facial landmark coordinates in terms of x and y values. Our proposed system used the dlib pre-trained model to detect facial landmarks on real-time images extracted from video frames (see Fig. 3).

### Eye detection, tracking, and blinking module

#### Eye detection and tracking module

The patient’s eyes are detected using the facial landmarks module, which locates the indexes of left and right eyes in the space of 68- (x, y) coordinates. Each eye is detected by 6 coordinates drawn on the created black mask of the same dimension for each reading frame corresponding to a patient’s image. The only white region on the created black mask surrounds the eye and expands using dilate morphological operation. The eyes are segmented through the bitwise-and operation with the created mask on the original image. All zeros pixel values are converted to 255 to localize the eyeball, the only black region left on the mask. The resulting eye mask is converted into grayscale to prepare the image for segmenting the eyeball from the eye and locating its center. We used a fixed threshold to create a binary mask to find the largest contour containing the eyeball and segment it accordingly. After the eyeball is detected, its position (in terms of x and y coordinates) is computed and returned as three values mapped to three directions: left (1), right (2), and up (3). The eye-down state is not detected in this version of the Blink-To-Live application since it conflicts with an eye-blinking state.

#### Eye blinking module

**Figure 4:**
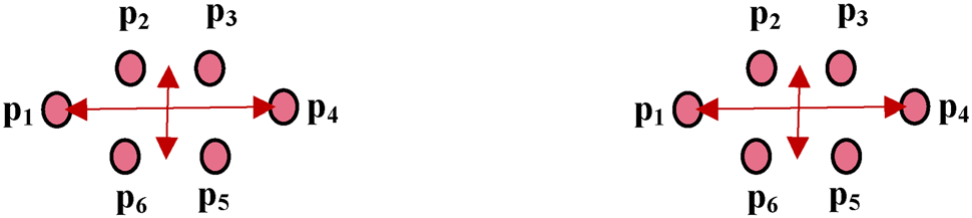
The 6-coordinates of left and right eyes, which are used in the Eye Aspect Ratio, EAR, computation.

Each eye is detected by 6 coordinates using the facial landmarks module, and the relation between the eye height and width can be encoded by the ratio called Eye Aspect Ratio, EAR^52,53^, that is computed by the following equation:

1 $${\varvec{E}}{\varvec{A}}{\varvec{R}}=\frac{\Vert {{\varvec{p}}}_{2}-{{\varvec{p}}}_{6}\Vert +\Vert {{\varvec{p}}}_{3}-{{\varvec{p}}}_{5}\Vert }{2\Vert {{\varvec{p}}}_{1}-{{\varvec{p}}}_{4}\Vert }$$

where p_1_, p_2_, p_3_, etc., are the coordinates of the eye’s landmarks, as depicted in Fig. 4.

$EAR$ is always constant when the eye is open, and it has an approximate value of zero when it blinks. Consequently, the ratio can determine whether the patient is blinking. A specific threshold value, $t$, is computed (i.e. 0.2 in our application). Suppose the *EAR* value is decreased by less than 0.2 and then increased above 0.2, a single blink is detected and can be counted as many as blinks have occurred. The following equation explains how the eye’s opening and closing states are detected based on comparing $EAR$ with the $t$ threshold value.

2 $$\left\{\begin{array}{cc} Eye\_closed& EAR \le t \\ Eye\_open& EAR >t\end{array}\right\}$$

The normal blink lasts from 100 to 400 ms, which is very fast compared to the intended blink (i.e., lasts 800 ms). We used the approach explained in^53^ to distinguish the normal blink from the patient’s blink, which is used as an alphabet in the Blink-To-Live eye-based language. The solution implies inspecting the $EAR$ value in the predefined number of video frames (i.e. 13 frames) and if the $EAR$ is still less than 0.2, then it’s the intended language blink. If the number of inspecting frames are less than 13 frames, the blink is very fast and might be a normal one considering that the camera captures 25 frames per second.

### Eye speech generation and translation module

From the previously discussed modules, the four eye alphabets (i.e., Blink (B), Left (L), Right (R), and Up (U)) can be detected and registered in an eye tracking list. The eye tracking list stores different eye-recognized states and generates the corresponding encoded phrases according to Tables 2 and 3. A python library *translate* is used to translate these phrases into different native languages such as Arabic, German, etc., according to different patients’ cultures and nationalities^54^. Further, the generated phrases text is converted to a lifelike synthesized speech using a Text-to-Speech module developed by Microsoft Azure^55^. The local registry keys of the Microsoft library for Text-to-Speech can also be used and imported directly into any python code.

### Blink-To-live system deployment

Blink-To-live is a flutter-based mobile application for helping patients with speech impairments to communicate with their families and caregivers. A mobile phone camera will be opened, and a stream of video frames will be captured and sent to a backend model via a web socket with FastAPI^56^. The two-way communication channel between a client (mobile application) and server (backend model) is established using a web socket with FastAPI that quickly sends images in real-time without going through all HTTP protocol layers. The web socket will efficiently handle a backpressure problem that is resulted from receiving more video frames than the expected number to be handled by the backend model. Also, this problem occurred when the model was busy processing existing frames and running face/eye detection modules, predicting the eye movements, and translating the recognized states into a communicating speech while receiving new frames from the mobile application. A queue or buffer with a limited size will be created to solve this problem. When the queue is full, some video frames will be dropped without affecting the application's efficiency since its details can be restored or created virtually from the previously stored ones. Two methods are implemented concurrently and running in parallel: *receive* and *process*. *Receive* method is used to read newly captured frames encoded by raw bytes. In contrast, the* process* method detects face/eyes in the previously received frames, tracks the eye gestures, and sends the translated eye’s speech back to the mobile application screen.

### Blink-To-Live system interaction with patients/caregivers

In this paper, we developed a mobile application called Blink-To-Live to help patients with speech impairments to communicate easily and efficiently. The patient’s caregiver only needs to open the mobile phone camera to capture and track the patient’s eye movements according to the Blink-To-Live four key alphabets: Left, Right, Up, and Blink. The patient’s communicated speech is generated according to the predefined dictionary of sentences encoded using a combination of three eye movement states. Once the sentences are synthesized, a translation module displays the phrases in the patient’s native language and the text-to-speech module generates a corresponding lifelike speech accordingly. Figure 4 shows different application screens, from the user’s registration to the screen that displays the eye movements encoded phrases.

**Figure 5:**
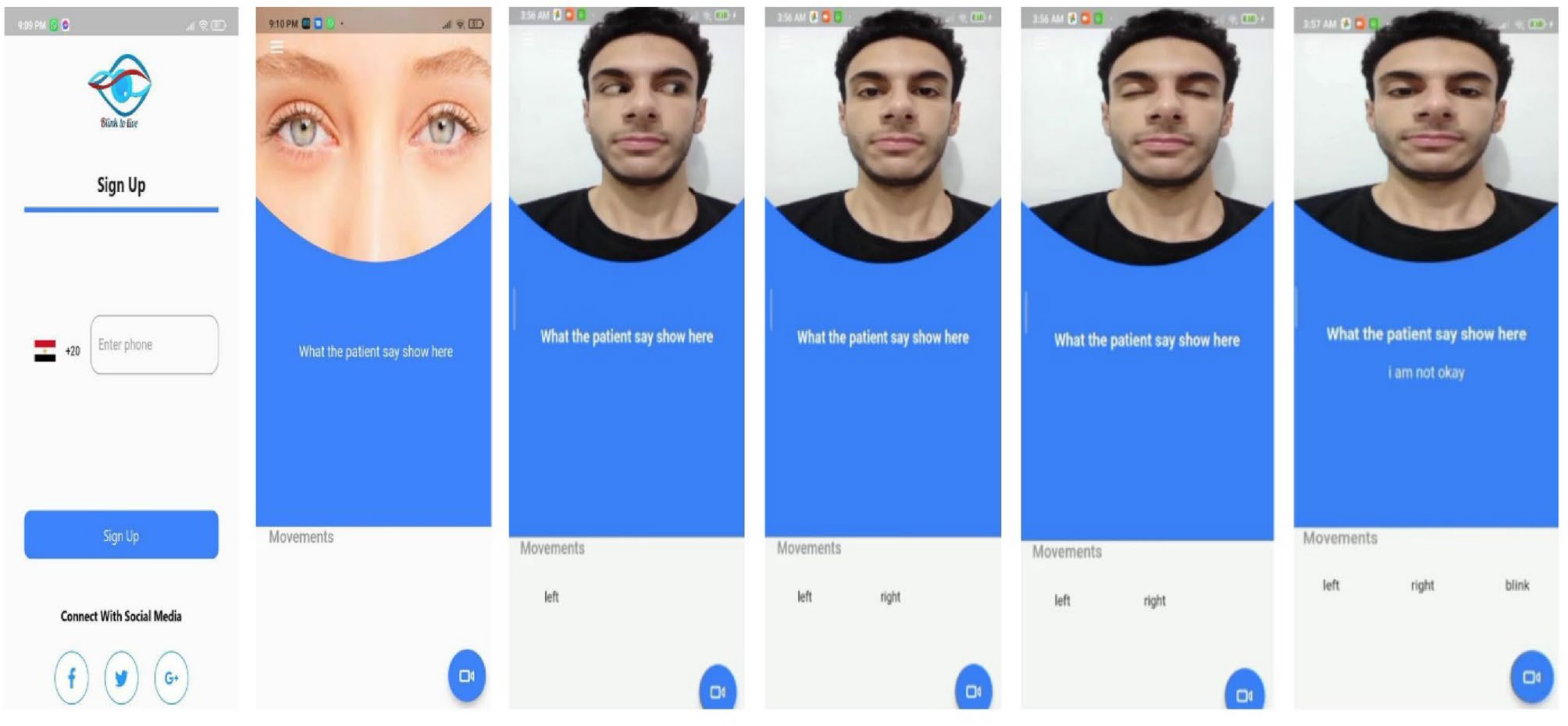
Blink-To-Live mobile application user interface (i.e., patient’s registration and eye tracking interfaces) along with the one displayed phrase “I am not Okay”.

In Fig. 5, users can register with their phone numbers and emails. Depending on the registration process, customized information, such as the patient’s nationality, culture, etc., can be inferred. The following application screens (i.e., Fig. 5) show that when the camera is opened, the application starts to track the eye movements and display them on the screen, and after all eye gestures are recognized, their corresponding synthesized phrase, according to Tables 2 and 3, is displayed on the application screen and its lifelike speech is heard.

**Figure 6:**
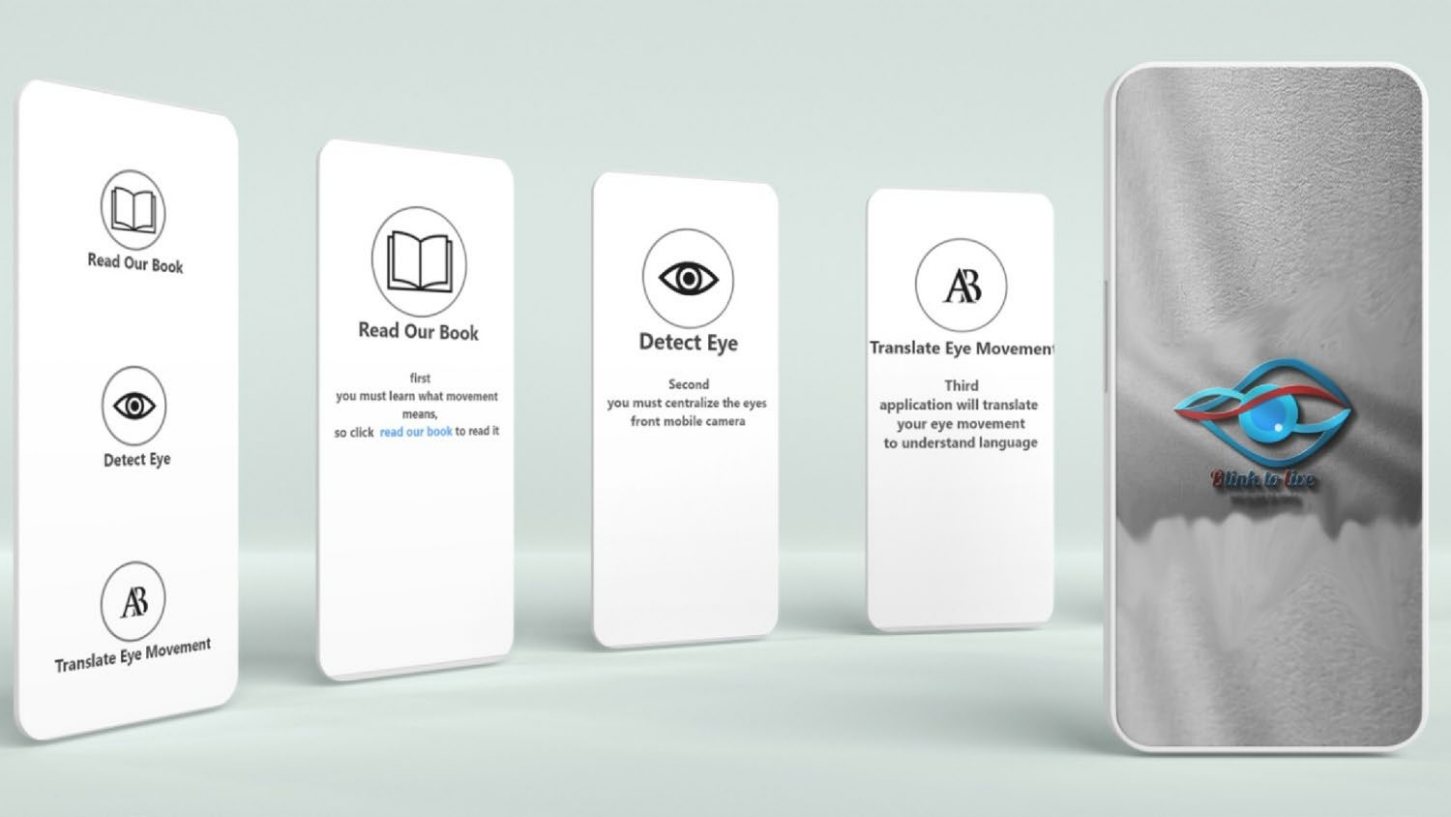
Blink-To-Live mobile application’s basic services: Reading the eye language book, Detecting the eye, and Translating the eye movements into phrases in the patient’s native speech.

**Figure 7:**
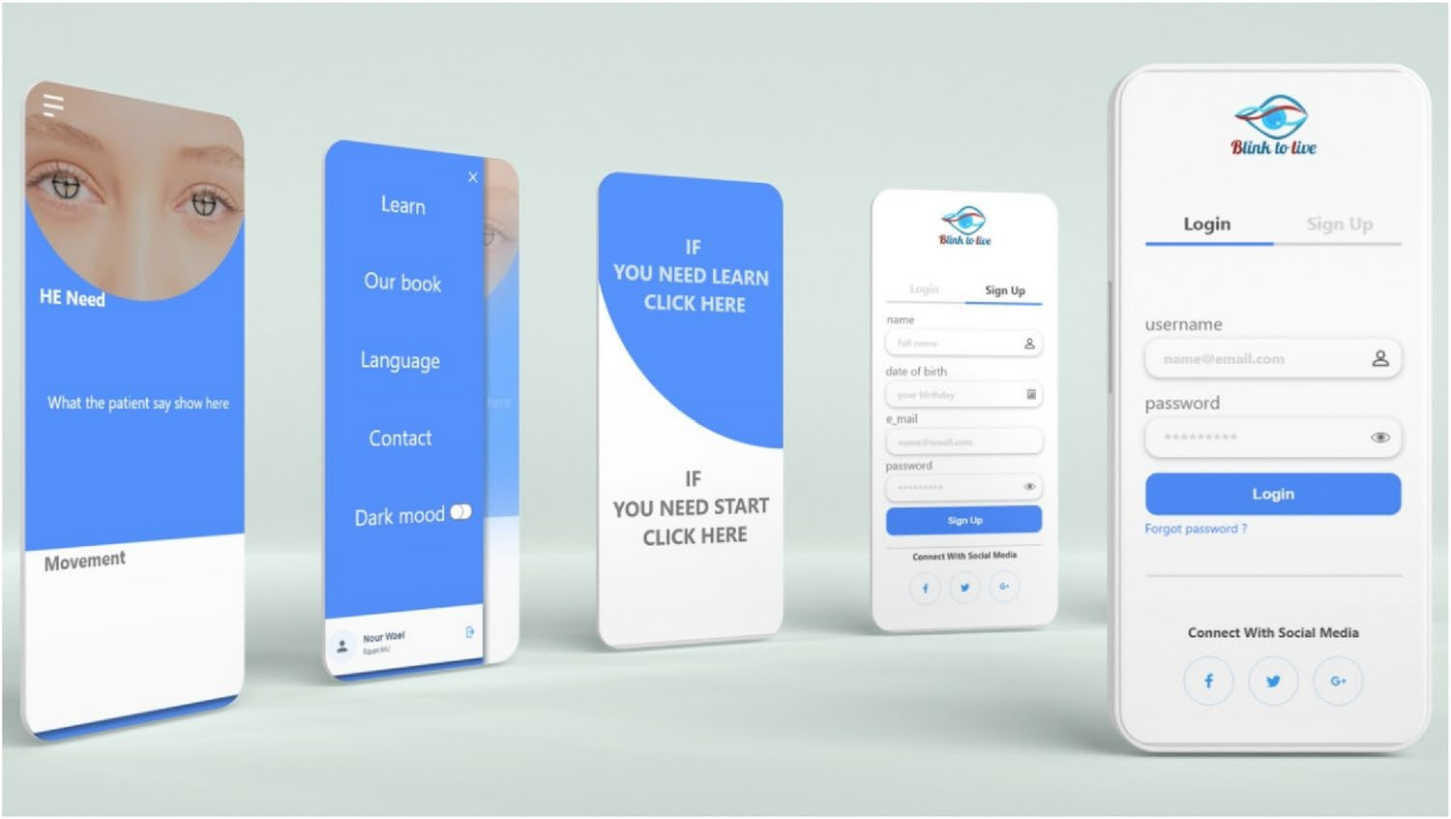
User Interface designs of the patient profile in the Blink-To-Live mobile application.

Also, the application has a learning module with animated graphs, which will help and motivate the patients and caregivers to learn more about the Blink-To-Live four eye movement alphabets and the corresponding eye-based generated speech. Further, the generated speech could be translated into different native languages according to the patient’s registration information (see Figs. 6 and 7).

**Figure 8:**
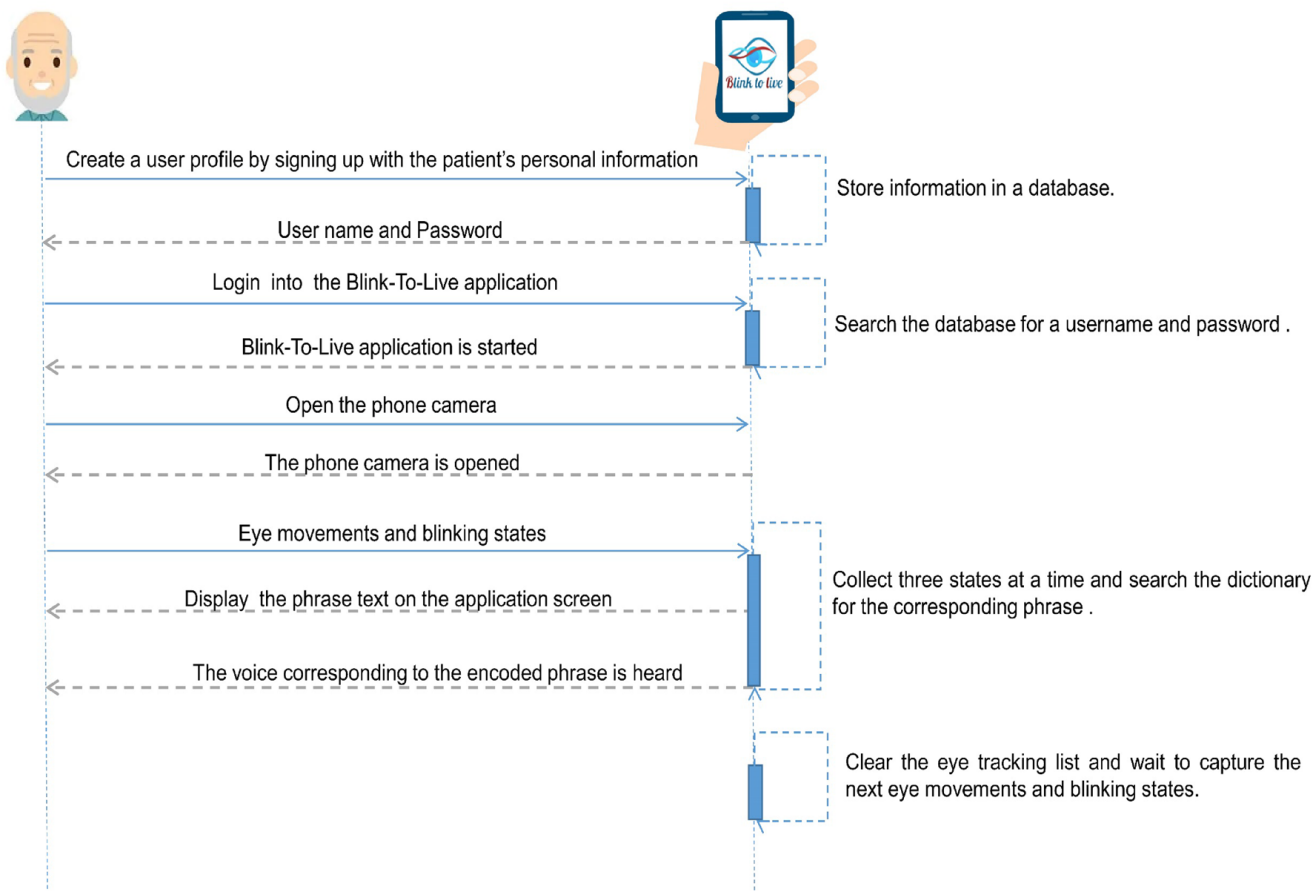
A sequence diagram that describes the interactions between the patient and the Blink-To-Live mobile application.

Figure 8 shows the interactions between the Blink-To-Live mobile application and the patient as a sequence of exchange messages. When a user profile is created for a patient by his caregiver, the login information can be used to start the Blink-To-Live application. Depending on the paralyzed stage, the patient or his caregiver can open the phone camera, and the application starts to track the patient’s eye and record each recognized movement in the eye tracking list. Every three recognized eye movements are translated into a corresponding phrase using the Blink-To-Live dictionary encoded by Tables 2 and 3. The encoded phrase is displayed on the application screen, and its corresponding voice is heard. The dictionary was encoded using three eye states to simplify communication without exhausting the patient’s eye by making a long sequence of eye movements. After the patient's encoded speech is displayed on the screen, the eye tracking list is cleared to start recording a new sequence of three eye gestures. Suppose the patient accidently makes an erroneous eye movement not recognized by the Blink-To-Live system. In that case, the application’s screen will not display the unrecognized state, and the patient will be assigned a five-second interval to express the correct one.

Different eye-tracking approaches are compared according to criteria such as communication speed, cost, caregiver dependency or special hardware devices for initiating the communication. Blink-To-Live follows an Indirect-eye contact tracking approach called a computer-vision-based eye-tracking approach. The comparison considered the results reported from different studies that evaluate different eye-tracking approaches for ALS patient communication^15,19,35,57,58^. The Blink-To-Live system does not rely on special hardware devices or sensors to initiate the patient’s communication. The patients/caregivers can use their mobiles with supported cameras to track the patient’s eyes, generate the intended speech, and translate it into the patient’s native language. No eyeglasses, electrodes, or gaze-sensing screens are needed; hence our application has the lowest cost compared to the other proposed ones. Blink-To-Live suffers from a small delay since extensive computer vision backend modules process the video frames in real-time and send the results back to the mobile application, so the communication speed will be slow compared to the direct-eye contact tracking systems (see Table 5).

**Table 5:** Comparing different approaches for eye-tracking according to some communication assessment criteria.

Assessment criteria	Direct-eye contact tracking	Indirect-eye contact tracking*	Eye tracking with electrical potentials
Caregivers are needed to assist the communication	No	No	No
Communication Speed	Fastest	Fast	Medium
Special devices/sensors are needed to initiate communications	Yes	No	Yes
Cost	Low	Lowest	Medium

*The proposed Blink-To-Live application follows this approach.

### Ethics declarations

All experiments were carried out by the relevant guidelines and regulations. Also, they were approved by the Scientific Research Ethics Committee, Faculty of Computers and Information, Mansoura University, Egypt. Also, the informed consent was obtained from all subjects to publish the information/image(s) in an online open access publication.

## Experimental results

A prototype of the proposed Blink-To-Live system has been tested using 10 normal cases with different demographic characteristics such as age, gender, educational level, and technology awareness. The technology awareness is evaluated according to the age, previous experience, cultural background, and the level of education of each participant included in the test experiment. Each case had one-week training to learn the different eye language alphabets and their related phrases in Tables 2 and 3. Then, each case is asked to speak 27 phrases by expressing their corresponding eye language alphabets (i.e., left, right, up, and blink). Each case had five trials to speak each one of the tested phrases.

Table 6 presents the recorded results, where each case had a coded symbol in the first column. The average communication speed, number of trials, and communication accuracy are reported in the following columns. The average communication speed is measured by the processing time required to recognize the eye’s gestures and display the corresponding phrase on the mobile screen. The communication accuracy is computed by counting the number of correctly spoken sentences using the eye alphabets out of the 27 tested sentences.

**Table 6:** Experimental results of Blink-To-Live mobile application.

Person ID	Educational level	Technology awareness	Communication speed in seconds	Communication accuracy	Trial number
P-01	High	High	15	26/27	1
P-02	High	High	20	27/27	1
P-03	Intermediate	Low	15	23/27*	2
P-04	High	High	10	26/27	1
P-05	Low	Low	25	10/27*	3
P-06	High	High	15	20/27	1
P-07	Intermediate	Low	23	19/27*	3
P-08	High	High	19	25/27	1
P-09	Intermediate	Low	24	20/27*	3
P-10	High	High	12	27/27	1

*These reported results are improved to 27, 20, 26, and 27, respectively, in one trial test after increasing the training period of these participants to 20 days.

Cases (P-01 to P-10) have 6 males and 4 females with ages ranging from 21 to 79. Cases P-01, P-02, P-04, P-06, P-08, and P-10 successfully express different eye language alphabets and correctly speak most of their related phrases in one trial (see Table 6). Cases P-01, P-02, P-04, and P-10, have younger ages compared to P-06 and P-08, with ages above 60. They all have a higher education level and technology awareness than the other participants. Cases P-03, P-07, and P-09, have intermediate educational level, and low technology awareness, so they needed more trials to express the eye language alphabets. Case P-05 has the worst performance as the educational level and technology awareness is very low compared to the others. The communication speed ranges from 15 to 25 s for expressing one sentence for all cases. This time will vary according to how many transition eye states are in the encoded phrase, the person’s ability to move their eyes correctly without moving their heads according to the intended speech, and the internet connection speed between the mobile application and the backend system. In our experimental results, normal people with high educational levels, technology awareness, good eye language training, good internet connectivity, and stability achieve good communication results. With sufficient training time, people with low educational level and technology awareness can achieve good performance. Patients with similar experimental settings will need more training (expected 15 days) to achieve the same performance. The patient’s dependency on his caregiver to open the phone camera relies on his ability to control his muscles according to different disease stages. Once the camera was opened to track the patient’s eye, there was no need for a caregiver to assist with the patient’s communication.

**Figure 9:**
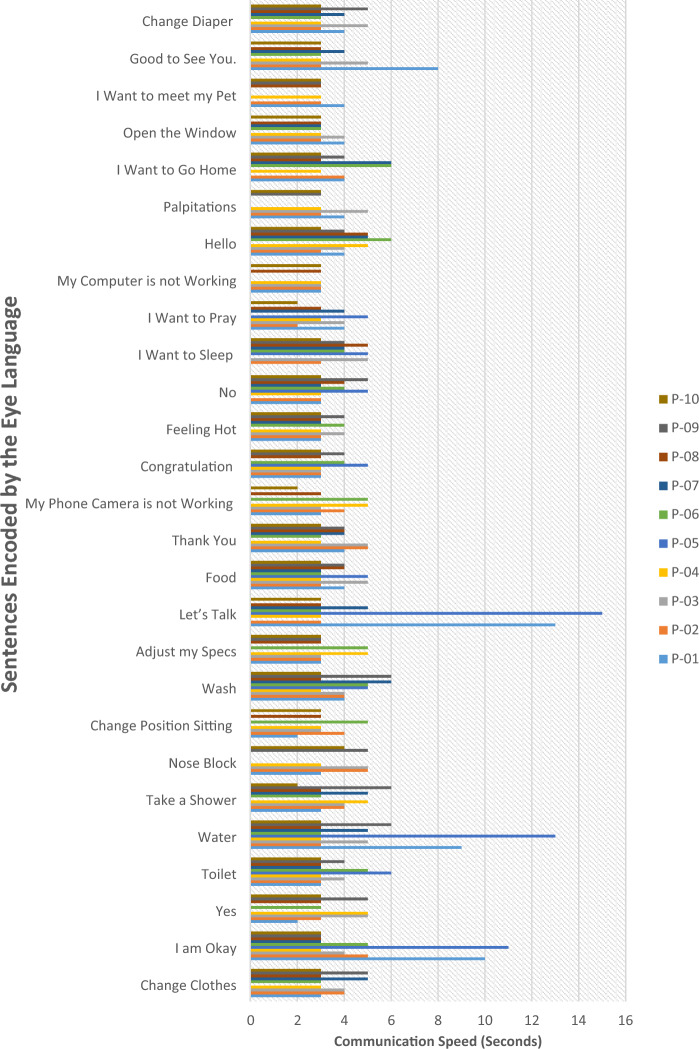
Blink-To-Live System communication speed of sentences with different transition eye states (i.e., L, R, U, and B).

To further evaluate the Blink-to-Live system, we removed any variable external factors such as the network speed/bandwidth, the total number of frames sent from a sender (i.e., mobile) to a server (backend model), frame resolution, and test the system using its desktop version. Overall, we reached an average time of 3 s for most of the communicated sentences with the same eye movement states or at least one transition state. Sometimes, blinking states take more time to recognize by our system, and accordingly, their corresponding phrases have a long communication time compared to the other phrases with no blinking states. Phrases such as “I Want to Sleep”, which is expressed by a sequence of [B L B], failed to be spoken by P-01 and P-04 since the system will not be able to recognize the third blinking state when a fast/slow transition was made between two different consecutive sates (see Fig. 9). Also, the sentences “Nose Block”, “Palpitations”,” My Computer is not Working”, and “I Want to Pray”, which are expressed by the consecutive sequence [L R R], [L R U], [U B R], [B R U], failed to be spoken by some participants as they made the fast transition between two consecutive states or moving their heads along with their eye’s gestures. Hence, the Blink-To-Live system cannot recognize the sequence of intended eye alphabets to decode its corresponding phrase.

Since Blink-To-Live communication speed is affected by many external factors such as the network speed/bandwidth, the total number of frames sent from mobile application to the server, and the frame resolution. The communication speed of the Blink-To-Live system, ${{\varvec{C}}{\varvec{S}}}^{{\varvec{B}}{\varvec{T}}{\varvec{L}}}$ , can be characterized mathematically as the following:

3 $${{\varvec{C}}{\varvec{S}}}^{{\varvec{B}}{\varvec{T}}{\varvec{L}}}={{\varvec{P}}}^{{\varvec{B}}{\varvec{T}}{\varvec{L}}}+{{\varvec{T}}}^{{\varvec{B}}{\varvec{T}}{\varvec{L}}}$$

where ${{\varvec{P}}}^{{\varvec{B}}{\varvec{T}}{\varvec{L}}}$ is the processing time of the sequence of three eye states on the server side and ${{\varvec{T}}}^{{\varvec{B}}{\varvec{T}}{\varvec{L}}}$ is the transmission time between the mobile application and the server.

On average, ${{\varvec{P}}}^{{\varvec{B}}{\varvec{T}}{\varvec{L}}}$ takes from three to four seconds, while ${{\varvec{T}}}^{{\varvec{B}}{\varvec{T}}{\varvec{L}}}$ can be defined in terms of the variables: ${D}^{BTL}$: duration time of the inspected video clip for patient’s eye movements,${FR}^{BTL}$: frame rate, ${F}^{BTL}$: total number of frames will be sent from the mobile application to the backend system, ${R}^{BTL}$: frame resolution characterized by a phone camera, ${X}^{BTL}$: number of pixels in video frames, ${B}^{BTL}$ : number of bits in video frames, and $N$: network bandwidth in bps.

4 $${F}^{BTL}= {D}^{BTL}\times {FR}^{BTL}$$

5 $${X}^{BTL}= {F}^{BTL}\times {R}^{BTL}$$

6 $${B}^{BTL}= {X}^{BTL}\times 8$$

7 $${T}^{BTL}= \frac{{B}^{BTL}}{N}$$

In the technical term, we compared two features-extractor approaches implemented in the dlib library. The first approach relies on CNN and has an execution time of 3.33 s for only a face detection module, while the Blink-To-Live-based approach has an execution time of 0.21 s (see Table 7).

**Table 7:** The execution time of the face detection module implemented by two different features-extractor approaches: CNN and HOG + SVM.

Model	CNN	HOG + SVM*
Execution time of face detection module	3.33 s	0.21 s

*The proposed Blink-To-Live application follows this approach.

Eye-based tracking applications such as Blink-To-Live have been introduced as assistive communication technology for patients with speech impairments. Two elements play a key role in the success of eye-based tracking technologies: the hardware device used to track the patient’s eye and the software application used to process the collected data. The devices that track the patient’s eye range from expensive gaze-sensing keyboards such as Tobii Dyanvox or Eye Transfer to special lenses, electrodes, or glasses with infrared sensors. The proposed Blink-To-Live system follows another approach for tracking the patient’s eye using a simple camera without using expensive specialized devices or hardware sensors directly positioned to the human eyes.

The software applications that process the collected data rely on the machine and deep learning techniques for facial landmarks detection, eye identification, and tracking. The Blink-To-Live system used HOG + SVM, the feature extractor approach implemented in the dlib library, for facial landmarks identification and other computer vision modules for tracking different eye movements and blinking states. The eye-down state is not detected in this version of the Blink-To-Live application since it conflicts with an eye-blinking state, which can be resolved in the future by using techniques such as reinforcement learning. Also, the Blink-To-Live system suffers from a small delay since extensive computer vision backend modules process the video frames in real-time and send the results back to the mobile application so that the communication speed will be slow compared to the other direct-eye contact tracking systems. Communication speed can be enhanced by developing fast processing backend services and web socket communication between the client and server applications.

## Conclusion

Blink-To-Live is a simple and cost-efficient mobile application for speech-impairment patients who only have their eyes to initiate communication with their surrounding world. It relies on a set of computer vision modules and a modified version of the Blink-To-Speak language to translate different eye gestures into a set of daily life commands used by the patients to express their emotions and needs. The patients/caregivers will only use their phones with supported cameras to track different patients’ eye movements. The synthesized eye-based speech will be displayed on the phone screen accordingly. The future improvements of our system are to increase communication speed by developing a fast processing backend system that utilizes modern real-time image analysis and processing approaches. Also, reinforcement learning algorithms can enhance eye detection, blinking, and tracking modules to achieve high-accuracy results and resolve conflicts among eye movement states. Further, each patient has a registered profile in our application. In the future, the application will learn his eye’s attributes, movements, and blinking behavior, which can increase the communication speed, and customize the application according to different patient needs. The Blink-To-Live could be integrated easily with other systems relying on hardware devices to control the patient’s living environment with eye gestures.

## Data Availability

Blink-To-Live is a free, open-source software released under the GNU GPL license and its source is the GitHub repository (https://github.com/ZW01f/Blink-To-Live).
